# Pheochromocytoma, “the Great Masquerader,” Presenting as Severe Acute Decompensated Heart Failure in a Young Patient

**DOI:** 10.1155/2018/8767801

**Published:** 2018-04-29

**Authors:** Hans A. Reyes, Jason J. Paquin, David M. Harris

**Affiliations:** ^1^Division of Cardiovascular Health and Disease, University of Cincinnati College of Medicine, Cincinnati, OH, USA; ^2^Division of Cardiology, Mercy Heart Institute, Cincinnati, OH, USA

## Abstract

A 22-year-old man presented to the hospital with progressive shortness of breath, chest discomfort, sinus tachycardia, and emesis. The echocardiogram demonstrated global hypokinesis with a left ventricle ejection fraction of 15–20%. The patient was treated for acute systolic heart failure decompensation with diuresis and afterload reduction. Unexpectedly, an abdominal computed tomography showed a left adrenal mass and subsequent serum/urine metanephrine tests suggested pheochromocytoma. Once the patient had stabilized, he underwent an uneventful adrenalectomy with histology results confirming the diagnosis of pheochromocytoma. After six months follow-up, he is currently doing well with close outpatient follow-up by cardiology.

## 1. Introduction

Pheochromocytoma, the “great masquerader,” is a neoplasm of neuroectodermal chromaffin cells which produces excess catecholamines. The classical clinical triad is described with headaches, palpitations, and sweating. However, this tumor can be asymptomatic or present with a myriad of symptoms including headaches, anxiety attacks, hypertension, and in rare cases, multiorgan failure [1–3]. These symptoms are secondary to excess catecholamines. Once diagnosed, the medical management can be further challenging. Our patient initially presented with severe cardiomyopathy and decompensated heart failure which required optimization prior to adrenalectomy.

## 2. Case

**Figure 1:**
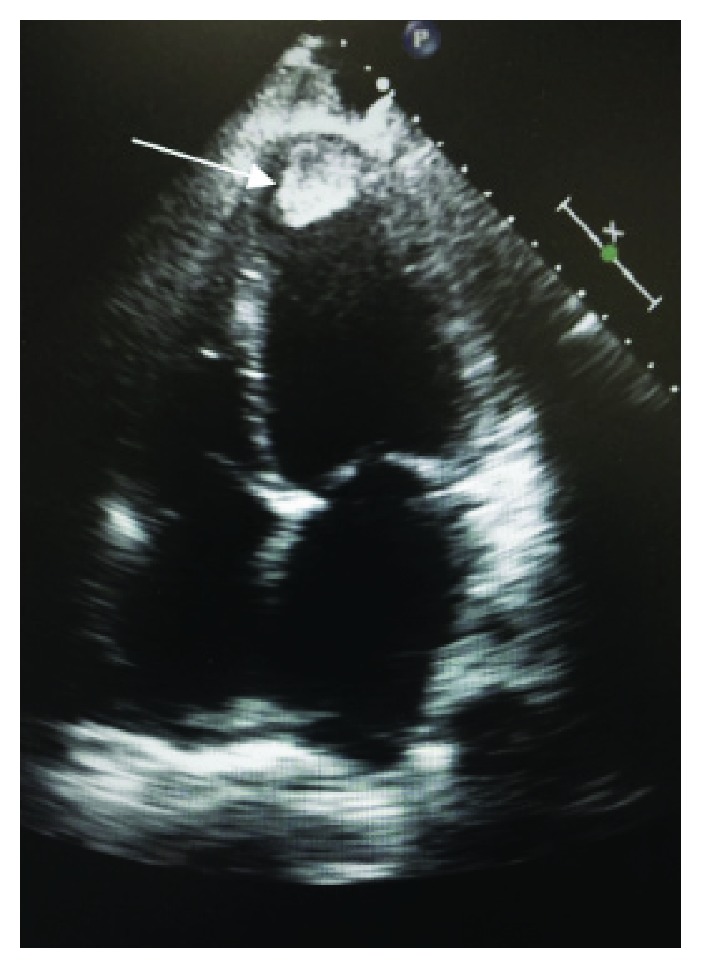
Echocardiogram: an apical four-chamber view showing a 2.2 × 1.4 cm left ventricular thrombus.

**Figure 2:**
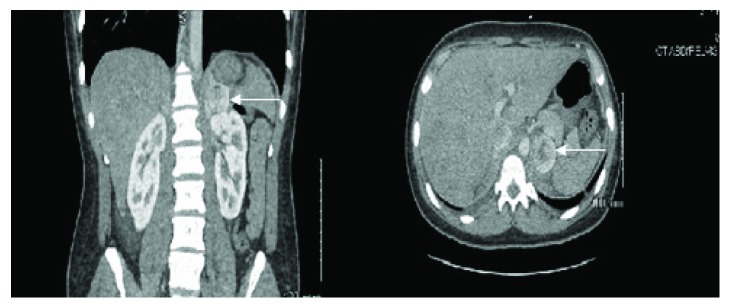
Abdomen/pelvic CT with contrast, coronal, and axial views showing the 4 × 3 cm left pheochromocytoma.

A 22-year-old male presented to the emergency department (ED) complaining of shortness of breath and abdominal swelling that had progressed over the previous two months. His symptoms were associated with intermittent chest discomfort and palpitations. Upon developing increased nausea, emesis, and diarrhea, he presented to the ED. The patient reported a history of hypertension and intermittent severe headaches but stated he did not seek additional evaluation or treatment. He had no history of tobacco or recreational drugs but reported occasional alcohol consumption. Initial vital signs included blood pressure 110/70 mmHg, heart rate 114 bpm, respiratory rate 18 per minute, O_2_ sat of 99% on room air, and temperature of 98°F. The physical exam was remarkable for a jaundiced patient in mild distress with jugular venous distention, tachycardia, and S3 gallop. Electrocardiogram confirmed sinus tachycardia with anterior ST depression. Laboratory data revealed the following: WBC 9800/*μ*L, Hb/Hct 14.5/45 g/dL, normal electrolytes, BUN/Cr 13/0.8, hepatic function was remarkable for ALT 153 mg/dL and AST 85 mg/dL, total bilirubin 3.9 mg/dL, normal coagulation profile, and troponin I < 0.01 ng/mL. The chest X-ray was notable for an enlarged cardiopericardial silhouette and prominent central pulmonary vasculature. The echocardiogram revealed severe global hypokinesis with left ventricular ejection fraction (LVEF) of 15–20%, apical akinesis with an organized 2.2 × 1.4 cm fixed thrombus (Figure 1), and a right ventricular systolic pressure estimated at 55 mmHg. A computed tomography (CT) pulmonary angiogram excluded pulmonary embolism but did show cardiomegaly and a right pleural effusion. An abdominal and pelvic CT with contrast was ordered due to gastrointestinal symptoms, and unexpectedly, it showed a 4 × 3 cm enhancing mass on the left adrenal gland (Figure 2).

The patient was admitted with a diagnosis of acute decompensated heart failure; the therapy included diuresis and afterload reduction with lisinopril, carvedilol as tolerated, and digoxin. Due to the suggestion of left ventricular thrombus by the echocardiogram, anticoagulation was initiated. A single-photon emission computed tomography (SPECT) stress test was done revealing severe global hypokinesis with regional variability, LVEF of 17%, and no evidence of ischemia. Incidentally, increased uptake associated with the left adrenal gland was noted. Plasma and 24-hour urine metanephrines were elevated at 11.8 nmol/L (normal value: 0–0.89 nmol/L) and 2223 *μ*g/24 h (normal value: 0–400 *μ*g/24 h), respectively, supporting the diagnosis of pheochromocytoma. Once heart failure condition was stabilized, carvedilol was switched to prazosin and metoprolol. The patient was transferred to a facility with cardiothoracic surgical support capabilities including left ventricular-assisted device and cardiac transplant prior to the planned adrenalectomy. Surgery was completed successfully with balloon pump backup and with histology results confirming the diagnosis of pheochromocytoma.

**Figure 3:**
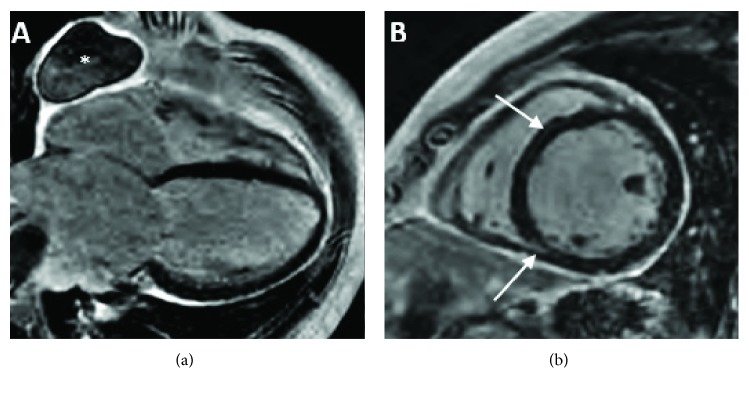
(a) CMRI showing the resolution of the left ventricular thrombus and an incidental pericardial cyst (asterisk). (b) CMRI showing slight delayed gadolinium enhancement with a left midventricular, midmyocardial septal stripe (superior arrow) and also an inferior right midventricular insertion delayed gadolinium enhancement (inferior arrow).

Subsequently, a cardiac magnetic resonance imaging (CMRI) performed two weeks after discharge showed moderate dilation of the left ventricle with a LVEF of 23%, diffuse hypokinesis, resolution of the intracavitary left ventricular thrombus, and a midventricular, midmyocardial linear stripe and inferior right midventricular insertion enhancement on the delayed gadolinium enhancement (DGE) (Figures 3(a) and 3(b)). Six months after adrenalectomy, the patient was able to return to work doing light activities four hours daily and his last echocardiogram showed an improved LVEF of 40%.

## 3. Discussion

Our case of a young male patient with a history of hypertension who presented with congestive heart failure highlights the importance of screening for secondary etiologies of hypertension in this population. While heart failure is uncommon in this population, the differential diagnosis includes viral myocarditis, drug-induced cardiomyopathy, familial cardiomyopathies, cardiac sarcoidosis, congenital heart disease, and, even rarer, ischemic cardiomyopathy.

Our patient had a history of hypertension for two years, was noncompliant, and was lost to follow-up. His acute presentation with heart failure was initially attributed to an uncontrolled hypertension. However, during the initial work-up, an abdominal CT showed a mass in the left adrenal gland with incidental radionuclide uptake during cardiac stress testing. Based on these findings, pheochromocytoma was suspected, which was subsequently confirmed by ancillary tests and pathology.

Pheochromocytoma, known as the “great masquerader,” is a neoplasm of neuroectodermal chromaffin cells which produces excess catecholamines. Its annual incidence is approximately 0.8 per 100,000 person-years and probably occurs in less than 0.5 percent of patients with hypertension [1, 2]. Pheochromocytoma can be sporadic or familial; sporadic forms are usually diagnosed between the ages of 40 and 50, while the hereditary forms present in childhood or early adulthood [3]. This tumor has varied clinical manifestations ranging from an asymptomatic subclinical picture to symptoms such as headaches, palpitations, sweating, anxiety, emesis, hypertension, and, in rare cases, multiorgan failure [4–7]. All of these symptoms are secondary to excess catecholamines. Importantly, relatively large numbers of patients are not diagnosed during their lives or are diagnosed only after the occurrence of significant cardiovascular damages [4], as with our patient.

Although the majority of patients with pheochromocytoma have a normal or increased ventricular systolic function on echocardiography, the pattern of left ventricular dysfunction may be variable. Only around 10–20% of patients with pheochromocytoma present with catecholamine-induced cardiomyopathy [8, 9]. When catecholamine-induced cardiomyopathy is present, most of the cases demonstrate a stress-induced cardiomyopathy, Takotsubo syndrome [7, 10]. In those patients, an echocardiogram demonstrates transient left ventricular apical ballooning with midventricular dyskinesis that extends beyond the distribution of any single coronary artery. Unlike stress-induced cardiomyopathy, our patient's echocardiogram demonstrated global hypokinesis and not apical ballooning which was initially suspected to be a consequence of occult hypertension leading to a “burned-out” ventricle.

Pheochromocytomas have been associated with other various cardiovascular complications due to overwhelming catecholamine levels. The most common complications include left ventricular hypertrophy, ischemic heart disease, disturbances in rhythm and conduction, malignant ventricular arrhythmias, and even shock [3, 5, 11]. The potentially fatal cardiovascular complications of these tumors are due to the potent effects of catecholamines, especially noradrenaline, the main transmitter released from sympathetic nerve terminals.

According to Batisse-Lignier et al. [7], which performed a systematic review in 145 cases with either acute or chronic pheochromocytoma-induced cardiomyopathy, the LVEF recovery after surgical resection of the tumor is higher in patients who presented with stress-induced cardiomyopathy than in others who presented with catecholamine-induced cardiomyopathies (64.9% versus 40.8%, *p* = 0.005). Even after a multivariate analysis, only stress-induced cardiomyopathy was associated with a better left ventricle recovery (*p* = 0.03).

The exact mechanism of cardiomyopathy in patients with pheochromocytoma remains unclear. However, there are some mechanisms that can explain the myocardial damage associated with catecholamines. These can cause a direct toxic effect on the myocardium through calcium overload due to altered sodium and calcium transporters, enhanced lipid mobility, free radical production, or increased sarcolemmal permeability [5, 10, 12]. In addition, myocardial damage may occur secondary to a decrease in myocardial oxygen supply combined with a sustained myocardial oxygen demand [12, 13]. Usually, catecholamines bind to beta1 and beta2-adrenergic receptors and produce positive inotropic and lusitropic effects. Nevertheless, in situations of increased stress with very high levels of catecholamines, beta1-adrenergic receptors' activation can induce apoptosis of the cardiac myocytes. Moreover, the binding of epinephrine to beta2-adrenergic receptors produces a negative inotropic effect [5, 10]. Thus, catecholamines also have a dose-dependent cardiotoxic effect.

Considering the previous mechanisms of cardiac injury, the myocardial damage produced in acute or chronic exposure to catecholamines could be different. Acute catecholamine exposure, such as in stress-induced cardiomyopathy, exposes the myocardial beta receptors to an enormous amount of catecholamines leading to a stunning of the left ventricle and slight histological apoptosis. On the other hand, in chronic adrenergic exposure, it is hypothesized that the heart gets gradually used to the surge of catecholamines with desensitization of adrenergic receptors [7]. Unlike acute exposure, long-standing catecholaminergic burden leads to structural myocardial alterations such as interstitial fibrosis, inflammation, and a subsequent dysfunction [10, 13]. This chronic exposure may explain the lower level of LVEF recovery in the catecholamine-induced cardiomyopathy subgroup described by Batisse-Lignier et al.

Previous reports have showed that pheochromocytoma-induced cardiomyopathy can be reversed with medical therapy first followed by a subsequent tumor extirpation [4, 6–9], as with our patient. The prognosis of catecholamine-induced cardiomyopathy depends on early diagnosis and prompt medical and surgical treatment. Once diagnosed, the medical management of these tumors prior to surgery may be challenging in the context of heart failure [14]. The usual therapy includes initial blockade of alpha-adrenergic receptors [8, 9]; beta-blockers are added after several days to ensure adequate alpha-adrenergic blockade. Beta blockers should never be administered without appropriate alpha blockade as this could worsen hypertensive episodes by exacerbating vasoconstriction while inhibiting vasodilation. Our patient had clear symptoms of decompensated heart failure on presentation, requiring reduction of both preload and afterload. Thus, we believe that the use of carvedilol, an alpha- and beta-adrenergic receptor blocker, was a rational and adequate option due to the possibility of pheochromocytoma. An alpha blocker is not a recommended treatment for heart failure, but its use in patients with pheochromocytoma is mandatory. Patients with pheochromocytoma may be intravascularly volume depleted, and diuretics should be administered with caution. However, in patients with catecholamine-induced cardiomyopathy, diuretic use is warranted. Regarding ancillary tests, interpretation of metanephrine levels in the context of established cardiomyopathy is also difficult as cardiac failure of any etiology generates an adrenergic response [14]. In this particular presentation of heart failure, where the culprit is an uncommon cause, multidisciplinary management is needed, including physiologically directed pharmacologic therapy which may deviate from guidelines given the need for alpha blockers and individualized care.

Our patient's CMRI showed minimal DGE consistent with mild myocardial fibrosis or scar, which was encouraging for left ventricular functional recovery. At six months follow-up, the patient is doing well, without significant symptoms, and has shown improvement of LVEF by echocardiogram. Given his young age at presentation with pheochromocytoma, he underwent genetic testing and is continuing to be followed by endocrinology.

With this case, we want to emphasize that the diagnosis of pheochromocytoma requires high clinical alertness due to its rare incidence and extremely variable clinical presentation and to highlight the relevance of considering pheochromocytoma in the differential diagnosis of unexplained cardiomyopathy in a young patient. We also want to stress the importance of routine follow-up in hypertensive patients as early diagnosis and appropriate treatment can significantly reduce morbidity and mortality in this group of patients. Finally, once diagnosed, it is essential to closely follow these patients to monitor the LVEF and provide optimal medical management for their cardiomyopathy.
